# The Typical Set and Entropy in Stochastic Systems with Arbitrary Phase Space Growth

**DOI:** 10.3390/e25020350

**Published:** 2023-02-14

**Authors:** Rudolf Hanel, Bernat Corominas-Murtra

**Affiliations:** 1 Complexity Science Hub Vienna, Josefstädter Strasse 39, 1080 Vienna, Austria; 2 Section for Science of Complex Systems, Medical University of Vienna, Spitalgasse 23, 1090 Vienna, Austria; 3 Institute of Biology, University of Graz, Holteigasse 6, 8010 Graz, Austria

**Keywords:** entropy, non-exponential phase space growth, typical set, asymptotic equipartition property, extensivity

## Abstract

The existence of the *typical set* is key for data compression strategies and for the emergence of robust statistical observables in macroscopic physical systems. Standard approaches derive its existence from a restricted set of dynamical constraints. However, given its central role underlying the emergence of stable, almost deterministic statistical patterns, a question arises whether typical sets exist in much more general scenarios. We demonstrate here that the typical set can be defined and characterized from general forms of entropy for a much wider class of stochastic processes than was previously thought. This includes processes showing arbitrary path dependence, long range correlations or dynamic sampling spaces, suggesting that typicality is a generic property of stochastic processes, regardless of their complexity. We argue that the potential emergence of robust properties in complex stochastic systems provided by the existence of typical sets has special relevance to biological systems.

## 1. Introduction

**Figure 1:**
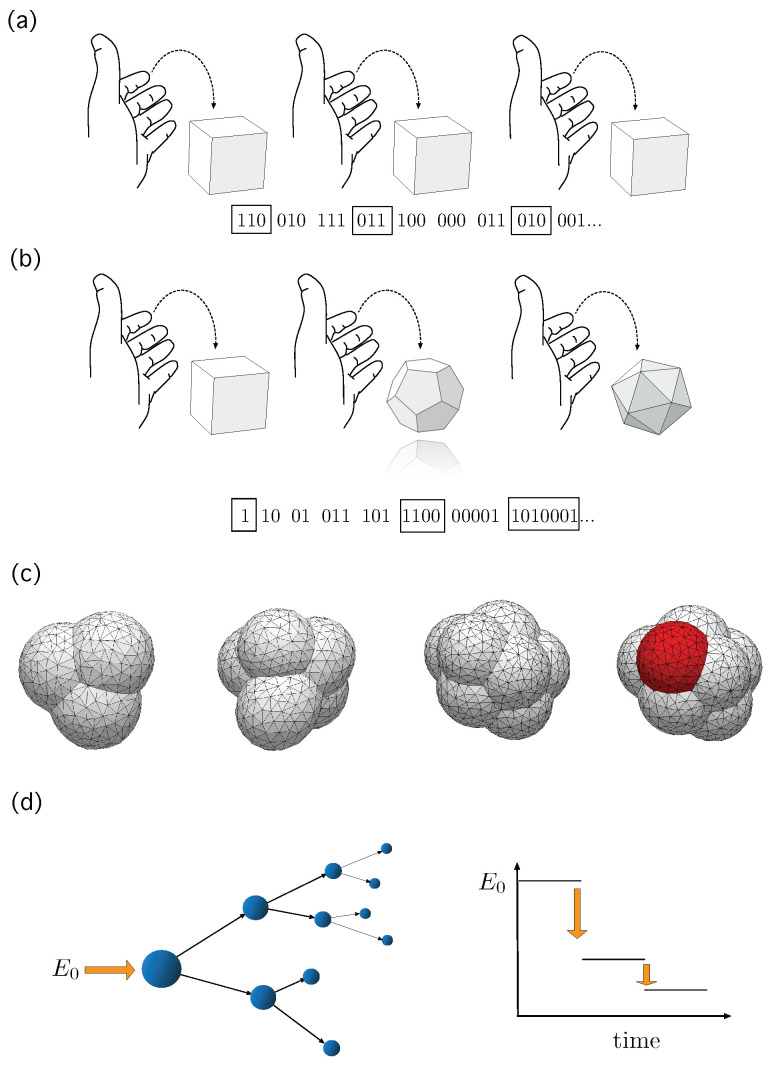
(**a**) Independent draws of the same dice, either fair or biased, define a i.i.d. stochastic processes whose typical set is well defined and grows following approximately an exponential trend [24]. (**b**) An example of a system whose typical set may show a super-exponential growth: At every drawing we update the dice by adding, e.g., a new face. (**c**) Potential configurations of early embryo development resembles, intuitively, the picture of the dice with growing number of faces. In this biological setting, new cells appear and, with that, new configurations but, on top of that, cells differentiate into new types—shown here in red—adding new states to the system that were not there before. Interestingly, even highly reproducible, the whole process displays a strong stochastic component [15]. (**d**) Nuclear disintegration can be studied from the framework of collapsing phase spaces [23]. In these processes, the amount of potential configurations of the system shrinks as long as the process unfolds. Toy models of embryo packings in (**c**) have been drawn using the evolver software package.

Many living systems are characterized by a high degree of internal stochasticity and display processes that form organization of growing complexity [1,2,3,4]. Such complexification processes exist on various scales, from the evolutionary scale [5,6], to the scale of single organisms [7]. The increase of complexity of the different forms of living entities triggered the debate whether the existence of *open-endedness* is a defining trait of biological evolution [8,9,10,11,12,13,14], with the resulting challenge of finding a potential statistical-physics-like characterization of it. At the single organism scale, the developmental process consists in the emergence of an adult multicellular organism from a single cell [7]. This astonishingly fast process of complexification sets challenges in many directions. One of these challenges is the characterization of the evolution of the space of potential configurations the system may acquire in time: In early embryo morphogenesis, for example, not only does the number of cells increase exponentially, resulting in the corresponding increase of potential configurations, but also cells differentiate into specialized cell types and create collective structures implying, in statistical physics language, that new states enter the system. This process is almost completely irreversible and, although highly precise, it is known to have a strong stochastic component [15,16,17]. Away from biology, non stable configuration spaces and processes of complexification have been identified in systems with innovation [18,19,20]. On the other hand, one can consider processes where the potential number of configurations decreases with time. Away from biology, recent advances in decay dynamics in nuclear physics were achieved considering a mathematical framework based on the stochastic collapse of the phase space [21,22,23]. In Figure 1 we schematically show the kind of processes we are exploring, all taking place in *dynamic phase spaces*, that is, processes where the amount of available possibilities may change (either grow or decrease) in time. Despite the ubiquity of such phenomena, a comprehensive characterization of systems with dynamic phase spaces, in terms equivalent to the ensemble theory of statistical mechanics, is lacking.

Ensemble formalism in statistical mechanics can be grounded in the concept of *typicality* [24,25,26,27,28]. Informally speaking, given the set of all potential sequences of events resulting from a stochastic process, a subset, the *typical set*, carries most of the probability [24,25]. This should not be confused with the set of most probable sequences: in the case of the biased coin, for example, the most probable sequence is not in the typical set. In other words, for long enough sequences, the probability that the observed sequence or state belongs to the subset of sequences forming the typical set goes asymptotically to 1. Generalizing to continuous systems, this is known as the *concentration of measure* phenomenon [29]. Accordingly, a typical property for a stochastic system is robust and acts as a strong, almost deterministic attractor as the process unfolds [27], and one may expect to observe it in the vast majority of cases. Moreover, if such a typical property exists, one can use this single property to—at least partially—characterize the system, and hence avoid a detailed microscopic description of all of the system’s components. Arguably, considerations based on typicality drive the connection between microscopic dynamics and macroscopic observables and underlie the existence of the thermodynamic limit [28,30,31]. In the context of information theory, the existence of the typical set for a given information source has deep consequences in the context of data compression [24,25].

The size of the typical set gives us valuable information on how the stochastic process is filling the phase space. In equilibrium systems or for information sources drawing independently from identically distributed (i.i.d.) random variables, the Gibbs–Shannon entropic functional arises naturally in the characterization of the typical set [24,25], establishing a clear connection between thermodynamics and phase space occupation. In systems/processes with collapsing or exploding phase spaces, path dependence or strong internal correlations [4,18,19,20,21,32,33,34,35,36,37], the phase space may grow super- or sub-exponentially, and the emergence of the Shannon–Gibbs entropic functional derived from phase space volume occupancy considerations is no longer guaranteed. The same situation may arise in cases dealing with non-stationary information sources [38,39,40,41,42]. Generalized forms for entropies have been proposed to encompass these more general scenarios [43,44,45,46,47,48,49,50,51], some of them explicitly linking the entropic functional to the expected evolution of the phase space volumes [36,46,52,53,54,55,56]. Despite the notable advances reported also for systems with physical significance [37,57,58], the concept of typicality has not been yet explored for systems/processes with exploding or shrinking phase spaces, which display path dependent dynamics or are subject to emergent internal constraints and correlations.

The purpose of this paper is to fill this important gap in the theory of stochastic processes, providing results with potential implications in the theory of non-equilibrium systems, data compression and coding strategies. As we shall see, the typical set can be defined for processes arbitrarily away from the i.i.d. framework, by only assuming a very generic convergence criteria, satisfied by a broad class of stochastic processes, that we refer to here as *compact stochastic processes*.

## 2. Results

### 2.1. Compact Stochastic Processes

Let us consider a general class of stochastic processes η [59,60]. This class encompasses almost any discrete stochastic process that can be conceived. A realization of *t* steps of the process is denoted as η(t):

$$\eta \left(t\right)=\eta_{1}\eta_{2}\ldots \eta_{t-1}\eta_{t},$$

where $\eta_{1},\eta_{2}\ldots ,\eta_{t-1},\eta_{t}$ are random variables themselves. Note that, in different realizations of *t* steps of the process, the sequence of random variables can be different, as the process may display path dependence, long term correlations, or changes of the state space dynamics itself (either shrinking or expanding). We denote a particular trajectory/path the process may follow as:

$$x\left(t\right)\equiv x_{1}x_{2}\ldots x_{t-1}x_{t}\in \Omega \left(t\right),$$

Ω(t) being the set of all possible paths of the process η up to time *t*. We focus on the family of stochastic processes where there exists (i) a positive, strictly concave and strictly increasing function $\Lambda \in C^{2}$ in the interval [1,∞), such that Λ(1)=0, and (ii) a positive, strictly increasing, $g\in C^{2}$, in the interval (1,∞), by which:

(1) $$\begin{array}{c} \lim_{t\to \infty }\frac{1}{g(t)}\Lambda \left(\frac{1}{p(\eta (t))}\right)=1, \end{array}$$

where the convergence is in probability [60]. We will call this family of stochastic processes *compact stochastic processes* (CSP). Given a CSP process η, a pair of functions Λ,g by which Equation (1) is satisfied define a *compact scale* of the CSP process η. Note that these two functions may not be unique for a given process, meaning that the process can, in principle, have several compact scales.

It is straightforward to check that, if η is a sequence of i.i.d. random variables $X_{1},\ldots ,X_{t}∼X$, Λ=log and g(t) is *t* times the Shannon entropy of a single realization, H(X), the above condition holds, as it recovers the standard formulation of the Asymptotic Equipartition Property (AEP) [24,25]. Therefore, the drawing of i.i.d. random variables ∼X is a CSP with compact scale (log,H(X)t). However, the range of potential processes that are CSPs is, in principle, much broader. In consequence, the first question we ask concerns the constraints that the convergence condition (1) imposes on Λ. Assuming that (1) holds, one finds that the candidates to characterize CSPs are the Λs satisfying the following condition; see Proposition A1 of the Appendix B.2 for details:

(2) $$\lim_{z\to \infty }\frac{\Lambda (\lambda z)}{\Lambda (z)}=1\;,\forall \lambda \in R^{+}.$$

Typical candidates for Λ are of the form $\Lambda \left(z\right)=c{\left(\log\left(z\right)\right)}^{d}$, where c,d are two positive, real valued constants or, more generally:

$$\Lambda \left(z\right)=c_{1}{\left(\log\left(1+c_{2}{\left(\log\left(1+c_{3}{\left(\log\left(\ldots \right)\right)}^{d_{3}}\right)\right)}^{d_{2}}\right)\right)}^{d_{1}},$$

where $c_{1},\ldots$ and $d_{1},\ldots$ are positive, real valued constants. In previous approaches, these constants have been identified as scaling exponents, enabling us to classify the different potential growth dynamics of the phase space [54]. We observe that the existence of the inverse function of Λ, $\Lambda^{-1}$, by which $\left(\Lambda^{-1}\circ \Lambda \right)\left(z\right)=z$, is guaranteed by the assumption made in the definition of CSPs that Λ is a strictly monotonically growing function.

The convergence condition defining CSPs has a direct consequence on how probabilities are distributed along the set of all potential paths. Indeed, from Equation (1) it follows that there exist two non-increasing sequences of positive numbers $\epsilon_{1},\ldots \epsilon_{t},\ldots$, $\delta_{1},\ldots \delta_{t},\ldots$, with $\lim_{t\to \infty }\epsilon_{t}=\lim_{t\to \infty }\delta_{t}=0$, from which one can define a sequence of subsets of paths $A\left[\epsilon_{1}\right]\ldots A\left[\epsilon_{t}\right]$ (such that $A\left[\epsilon_{1}\right]\subseteq \Omega \left(1\right),\ldots ,A\left[\epsilon_{t}\right]\subseteq \Omega \left(t\right)$) as follows: For all $x\left(t\right)\in A\left[\epsilon_{t}\right]$:

(3) $$\frac{1}{\Lambda^{-1}\left(\left(1+\epsilon_{t}\right)g\left(t\right)\right)}\leq p\left(x\left(t\right)\right)\leq \frac{1}{\Lambda^{-1}\left(\left(1-\epsilon_{t}\right)g\left(t\right)\right)}\;,$$

and the probability of a given path to belong to $A[\epsilon_{t}]$ is bounded as:

(4) $$P\left(x\left(t\right)\in A\left[\epsilon_{t}\right]\right)>1-\delta_{t},$$

where:

$$P\left(x\left(t\right)\in A\left[\epsilon_{t}\right]\right)=\sum_{x\left(t\right)\in A\left[\epsilon_{t}\right]}p\left(x\left(t\right)\right).$$

We call the sequence of subsets $A\left[\epsilon_{1}\right]\ldots A\left[\epsilon_{t}\right]$ of the respective sampling spaces Ω(1)…Ω(t) a *sequence of typical sets of η*. Informally speaking, Equation (4) tells us that, for large enough *t*, the probability of observing a path that does not belong to the typical set becomes negligible. As a consequence, *the typical set can be identified for CSPs*: Given a CSP, the typical set $A[\epsilon_{t}]$ absorbs all the probability, in the limit t→∞. We summarize the above considerations in a theorem:

**Theorem** **1.** *If η is a CSP in (Λ,g), then there exist (a) a non-increasing sequence of positive numbers $\epsilon_{1},\epsilon_{2},\ldots ,\epsilon_{t-1},\epsilon_{t},\ldots$ with limit $\lim_{t\to \infty }\epsilon_{t}=0$, associated to η and hence (b) the respective sequence of typical sets $A\left[\epsilon_{1}\right],\ldots ,A\left[\epsilon_{t}\right],\ldots$ by which:*

$$\lim_{t\to \infty }P\left(x\left(t\right)\in A\left[\epsilon_{t}\right]\right)=1.$$

**Proof.**  Since η is a CSP we know, by assumption, that, as t→∞ also Λ(1/p(η(t)))/g(t)→1 (in probability). We can rewrite this condition by stating that, for every ϵ,δ>0, there exists a $t_{0}$ by which, for each $t>t_{0}$ [60]:

$$P\left(\left|\frac{1}{g(t)}\Lambda \left(\frac{1}{p(x(t))}\right)-1\right|>\epsilon \right)<\delta .$$

Let τ(ε,δ) be the smallest such $t_{0}$. We can then use two arbitrary strictly monotonic decreasing functions $\epsilon_{n}^{*}$ and $\delta_{n}^{*}$ that converge to zero and construct a monotonically increasing sequence of times $t_{n}=\tau \left(\epsilon_{n}^{*},\delta_{n}^{*}\right)$ such that for all $t\geq t_{n}$ it is true that

$$P\left(\left|\frac{1}{g(t)}\Lambda \left(\frac{1}{p(x(t))}\right)-1\right|>\epsilon_{n}^{*}\right)<\delta_{n}^{*}.$$

From that, it is straightforward to define a non-increasing sequence $\epsilon_{1},\ldots ,\epsilon_{t},\ldots$ converging to 0, by just taking:

$$\left(\forall t:t_{n}\leq t<t_{n+1}\right)\;,\;\epsilon_{t}=\epsilon_{n}^{*}.$$

Finally, the condition:

$$\lim_{t\to \infty }P\left(x\left(t\right)\in A\left[\epsilon_{t}\right]\right)=1,$$

follows as a direct consequence of the construction of the sequence of typical sets $A[\epsilon_{1}],$$\ldots ,A[\epsilon_{t}],\ldots$, thereby concluding the proof. □

We omitted a direct reference to the process η in the notation of the typical set (i.e., $A\left[\epsilon_{t}\right]\equiv A\left[\epsilon_{t}\right]\left(\eta \right)$) for the sake of readability, and we will explicitly refer to it only if it is strictly necessary. In the next section we provide more details on the specific bounds in size by studying a subclass of the CSPs, namely, the class of *simple* CSPs. For them, the characterization of the typical set can be achieved using generalized forms of entropy.

### 2.2. The Typical Set and Generalized Entropies

Equation (1) can be related to a general form of path entropy:

(5) $$S_{\Lambda }\left(\eta \left(t\right)\right)=\sum_{x(t)\in \Omega (t)}p\left(x\left(t\right)\right)\Lambda \left(\frac{1}{p(x(t))}\right),$$

It can be proven that $S_{\Lambda }$ satisfies three of the four Shannon–Khinchin axioms expected by an entropic functional [25,61,62] in Khinchin’s formulation [62], to be referred to as SK1, SK2, SK3. In particular SK1 states that entropy must be a function of the probabilities, which is satisfied by $S_{\Lambda }$, by construction. SK2 states that $S_{\Lambda }$ is maximized by the uniform distribution *q* over Ω(t), i.e.,

$$q\left(x\left(t\right)\right)=\frac{1}{\left|\Omega (t)\right|}.$$

Finally, SK3 states that, if p(x(t))=0, then p(x(t)) does not contribute to the entropy, which implies:

$$\lim_{p(x(t))\to 0}p\left(x\left(t\right)\right)\Lambda \left(\frac{1}{p(x(t))}\right)=0,$$

satisfied as well for any Λ considered in the definition of the CSPs.

We further observe that $S_{\Lambda }$ is a monotonically increasing function as well, in the case of uniform probabilities: Let us suppose two CSPs η and $\eta^{\prime }$ that sample uniformly their respective sampling spaces, $\Omega \left(t\right),\Omega^{\prime }\left(t\right)$, such that $\left|\Omega \left(t\right)|<\right|\Omega^{\prime }\left(t\right)\left|\right)$. Let, in consequence, *q* and $q^{\prime }$ be the uniform distributions over Ω(t) and $\Omega^{\prime }\left(t\right)$, respectively. Then:

$$S_{\Lambda }\left(q\right)=\Lambda \left(|\Omega (t)|\right)<\Lambda \left(|\Omega^{\prime }\left(t\right)|\right)=S_{\Lambda }\left(q^{\prime }\right),$$

where $S_{\Lambda }\left(q\right),S_{\Lambda }\left(q^{\prime }\right)$ are the generalized entropies as defined in Equation (5) applied to distributions *q* and $q^{\prime }$. In the Proposition A3 of the Appendix C we provide details of the above derivations. We observe that SK4 is not generally satisfied: This axiom states that S(AB)=S(A)+S(B|A), and one can only guarantee its validity in the case of Shannon entropy, where Λ=log. In the general case, this condition may not be satisfied. A different arithmetic rule can substitute SK4 to accommodate other entropic forms [46]. Notice, however, that the use of Shannon (path) entropy, i.e., Λ=log, in the compact scale of a CSP may be used in in a broad spectrum of cases, including systems with correlations or super-exponential sample space growth, as we will see in Section 2.3.

If the contributions to the above entropy of the paths belonging to the complementary set of $A[\epsilon_{t}]$, that is, $\Omega \left(t\right)\setminus A\left[\epsilon_{t}\right]$, are negligible in the limit of t→∞, then we call the CSP *simple*. For simple CSPs with compact scale (Λ,g) the following convergence condition holds:

(6) $$\frac{S_{\Lambda }\left(\eta \left(t\right)\right)}{g(t)}\to 1.$$

Moreover, in simple CSPs, the typical set has the largest contribution to the entropy. First we define the contribution of the typical set to the entropy, $S_{\Lambda }\left(A\left[\epsilon_{t}\right]\right)$, as:

$$S_{\Lambda }\left(A\left[\epsilon_{t}\right]\right)=\sum_{x\left(t\right)\in A\left[\epsilon_{t}\right]}p\left(x\left(t\right)\right)\Lambda \left(\frac{1}{p(x(t))}\right).$$

We then demonstrate the above claim with the following proposition:

**Theorem** **2.** *If η is a simple CSP with compact scale (Λ,g), there exists a non-increasing sequence of positive numbers $\epsilon_{1},\epsilon_{2},\ldots ,\epsilon_{t-1},\epsilon_{t},\ldots$ with limit $\lim_{t\to \infty }\epsilon_{t}=0$ and its corresponding sequence of typical sets $A\left[\epsilon_{1}\right],\ldots ,A\left[\epsilon_{t}\right],\ldots$, such that:*

$$\lim_{t\to \infty }\frac{S_{\Lambda }\left(\eta \left(t\right)\right)}{g(t)}=\lim_{t\to \infty }\frac{S_{\Lambda }\left(A\left[\epsilon_{t}\right]\right)}{g(t)}=1.$$

**Proof.**  We will start with the second equality, namely:

$$\lim_{t\to \infty }\frac{S_{\Lambda }\left(A\left[\epsilon_{t}\right]\right)}{g(t)}=1.$$

From the definition of typical sets we know that, for paths $x\left(t\right)\in A\left[\epsilon_{t}\right]$, it is true that:

$$\frac{1}{\Lambda^{-1}\left(\left(1+\epsilon_{t}\right)g\left(t\right)\right)}\;\leq \;p\left(x\left(t\right)\right)\;\leq \;\frac{1}{\Lambda^{-1}\left(\left(1-\epsilon_{t}\right)g\left(t\right)\right)}.$$

In consequence, given that $P\left(x\left(t\right)\in A\left[\epsilon_{t}\right]\right))>1-\delta_{t}$, one can bound $S_{\Lambda }\left(A\left[\epsilon_{t}\right]\right)$ as:

$$\left(1-\delta_{t}\right)\left(1-\epsilon_{t}\right)<\frac{S_{\Lambda }\left(A\left[\epsilon_{t}\right]\right)}{g(t)}<\left(1-\delta_{t}\right)\left(1+\epsilon_{t}\right).$$

Since, by construction $\lim_{t\to \infty }\epsilon_{t}=\lim_{t\to \infty }\delta_{t}=0$, this second part of the theorem is proven. From that, the statement of the theorem:

$$\lim_{t\to \infty }\frac{S_{\Lambda }\left(\eta \left(t\right)\right)}{g(t)}=\lim_{t\to \infty }\frac{S_{\Lambda }\left(A\left[\epsilon_{t}\right]\right)}{g(t)}=1,$$

follows directly given the assumption of simplicity. □

In consequence, the typical set can be naturally defined for simple CSPs in terms of the generalized entropy $S_{\Lambda }$. To see that, we first reword condition (1) for simple CSPs as:

$$\lim_{t\to \infty }\frac{1}{S_{\Lambda }\left(\eta \left(t\right)\right)}\Lambda \left(\frac{1}{p(\eta (t))}\right)=1,$$

(in probability). We can rewrite the above condition in a more convenient form: Given a simple CSP η, there are two non-increasing sequences of positive numbers $\epsilon_{1},\ldots \epsilon_{t},\ldots$, $\delta_{1},\ldots \delta_{t},\ldots$, with $\lim_{t\to \infty }\epsilon_{t}=\lim_{t\to \infty }\delta_{t}=0$, by which:

(7) $$P\left(\left|\frac{1}{S_{\Lambda }\left(\eta \left(t\right)\right)}\Lambda \left(\frac{1}{p(x(t))}\right)-1\right|>\epsilon_{t}\right)<\delta_{t}.$$

Then, for each t>0 there is a set of paths, the *typical set* $A\left[\epsilon_{t}\right]\subseteq \Omega \left(t\right)$, such that for all $x\left(t\right)\in A\left[\epsilon_{t}\right]$:

$$\begin{array}{ccc} \frac{1}{\Lambda^{-1}\left(\left(1+\epsilon_{t}\right)S_{\Lambda }\left(\eta \left(t\right)\right)\right)} & \leq & p(x(t))\\ & \leq & \frac{1}{\Lambda^{-1}\left(\left(1-\epsilon_{t}\right)S_{\Lambda }\left(\eta \left(t\right)\right)\right)}, \end{array}$$

by which $P\left(x\left(t\right)\in A\left[\epsilon_{t}\right]\right)>1-\delta_{t}$. Notice that, now, the typical set is characterized using the generalized entropy $S_{\Lambda }$.

The next obvious question refers to the cardinality of the typical set $\left|A[\epsilon_{t}]\right|$. We will see that it can be bounded from above and below in a way analogous to the standard one [24]. The first bound is obtained by observing that:

$$\begin{array}{ccc} 1-\delta_{t} & \leq & \sum_{x\left(t\right)\in A\left[\epsilon_{t}\right]}p\left(x\left(t\right)\right)\\ & \leq & \frac{\left|A[\epsilon_{t}]\right|}{\Lambda^{-1}\left(\left(1-\epsilon_{t}\right)S_{\Lambda }\left(\eta \left(t\right)\right)\right)}, \end{array}$$

where $\Lambda^{-1}$ is the inverse function of Λ, i.e., $\left(\Lambda^{-1}\circ \Lambda \right)\left(z\right)=z$, which exists given the assumption that Λ is a monotonically growing function made in the definition of CSPs. From that, it follows that the cardinality of the typical set is bounded from below as:

(8) $$|A\left[\epsilon_{t}\right]|\geq \left(1-\delta_{t}\right)\Lambda^{-1}\left(\left(1-\epsilon_{t}\right)S_{\Lambda }\left(\eta \left(t\right)\right)\right).$$

For the upper bound, we observe that:

$$\begin{array}{ccc} 1 & \geq & \sum_{x\left(t\right)\in A\left[\epsilon_{t}\right]}p\left(x\left(t\right)\right)\\ & \geq & \frac{\left|A[\epsilon_{t}]\right|}{\Lambda^{-1}\left(\left(1+\epsilon_{t}\right)S_{\Lambda }\left(\eta \left(t\right)\right)\right)}, \end{array}$$

leading to:

(9) $$|A\left[\epsilon_{t}\right]|\leq \Lambda^{-1}\left(\left(1+\epsilon_{t}\right)S_{\Lambda }\left(\eta \left(t\right)\right)\right).$$

Given the bounds provided in Equations (8) and (9), one can (roughly) estimate the cardinality of the typical set as:

(10) $$|A\left[\epsilon_{t}\right]|\approx \Lambda^{-1}\left(S_{\Lambda }\left(\eta \left(t\right)\right)\right).$$

We present a rigorous version of the above result as a proposition.

**Proposition** **1.** *Let η be a simple CSP in (g,Λ) with some typical localizer sequence $\epsilon_{1},\ldots ,\epsilon_{t},\ldots$, then:*

$$\lim_{t\to \infty }\frac{\Lambda (|A\left[\epsilon_{t}\right]|)}{S_{\Lambda }\left(\eta \left(t\right)\right)}\;=\;1.$$

**Proof.**  From Equations (8) and (9), one can derive the following chain of inequalities:

$$\begin{array}{ccc} \Lambda (\left(1-\epsilon_{t}\right)\Lambda^{-1}\left(\left(1-\epsilon_{t}\right)S_{\Lambda }\left(\eta \left(t\right)\right)\right)) & < & \Lambda (|A\left[\epsilon_{t}\right]|)\\ & < & \left(1+\epsilon_{t}\right)S_{\Lambda }\left(\eta \left(t\right)\right). \end{array}$$

The last term poses no difficulties. To explore the behavior of the first one, we just rename the term:

$$z\equiv \Lambda^{-1}\left(\left(1-\epsilon_{t}\right)S_{\Lambda }\left(\eta \left(t\right)\right)\right),$$

and rewrite the first term of the inequality:

$$\Lambda \left(\left(1-\epsilon_{t}\right)\Lambda^{-1}\left(\left(1-\epsilon_{t}\right)S_{\Lambda }\left(\eta \left(t\right)\right)\right)\right)=\Lambda \left(\left(1-\epsilon_{t}\right)z\right).$$

We know, from Proposition A1, that the functions we are dealing with behave such that:

$$\lim_{z\to \infty }\frac{\Lambda (\lambda z)}{\Lambda (z)}\to 1\;,\;\left(\forall z>0\right).$$

As a consequence:

$$\frac{\Lambda (\left(1-\epsilon_{t}\right)\Lambda^{-1}\left(\left(1-\epsilon_{t}\right)S_{\Lambda }\left(\eta \left(t\right)\right)\right))}{S_{\Lambda }\left(\eta \left(t\right)\right)}\to 1.$$

Therefore, since also the third term goes trivially to $∼S_{\Lambda }\left(\eta \left(t\right)\right)$, we can conclude that:

$$\frac{\Lambda (|A\left[\epsilon_{t}\right]|)}{S_{\Lambda }\left(\eta \left(t\right)\right)}\;\to 1,$$

as we wanted to demonstrate. □

The above proven asymptotic equivalence gives us the opportunity of rewriting the entropy in a *Boltzmann-like* form:

$$S_{\Lambda }\left(\eta \left(t\right)\right)∼\Lambda (|A\left[\epsilon_{t}\right]\left|\right).$$

This identifies the cardinality of the typical set with Boltzmann’s *W*, the number of alternatives the system can effectively display: Finally, we notice that we can (roughly) approximate the typical probabilities as:

$$p\left(x\left(t\right)\right)\approx \frac{1}{\Lambda^{-1}\left(S_{\Lambda }\left(\eta \left(t\right)\right)\right)}.$$

We thus provided a general proof that the typical set exists and that it can be properly defined for a wide class of stochastic processes, the CSPs, those satisfying convergence condition (1). Moreover, we show that its volume can be bounded and fairly approximated as a function of the generalized entropy emerging from the convergence condition, $S_{\Lambda }$, as defined in Equation (5).

### 2.3. Example: A Path Dependent Process

**Figure 2:**
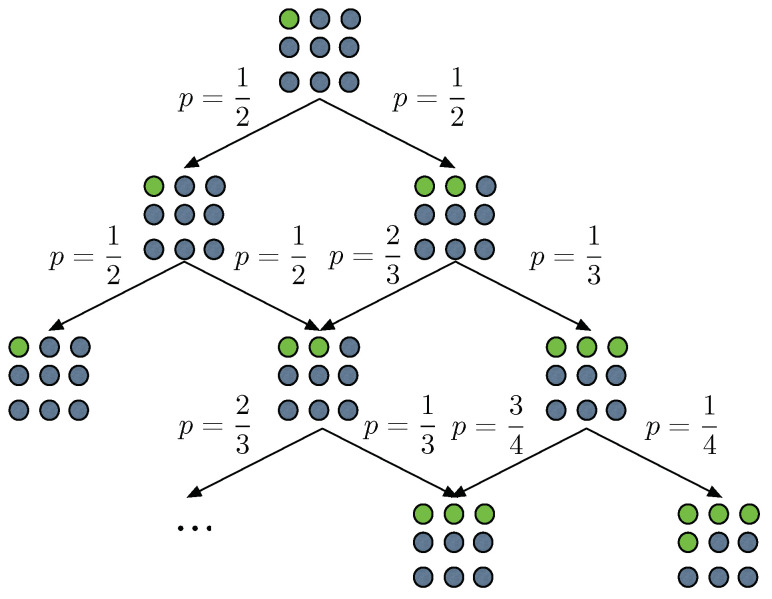
The rules of the Chinese restaurant process with memory. Here green circles represent occupied tables and grey circles empty table. Notice that in the mathematical formulation of the problem the number of tables is infinite. Arrows depict the possible transitions of the process and the associated probabilities.

We briefly explore the behavior of the typical set and its associated entropic forms through a model displaying both path dependence and unbounded growth of the phase space. The process η works as follows: Let us suppose we have a restaurant with an infinite number of tables $m_{1},\ldots .,m_{n},\ldots$. At $t_{0}=0$ a customer enters the restaurant and sits at table $m_{1}$. At time *t* a new customer enters the restaurant where already m(t) tables are occupied; the occupation number of each table is unbounded. The customer can chose either to sit at an already occupied table from the $m_{1},\ldots ,m_{m(t)}$ occupied tables, each with equal probability $\frac{1}{m(t)+1}$, or in the next unoccupied one, $m_{m(t)+1}$, again with probability $\frac{1}{m(t)+1}$. This process is a version of the so-called *Chinese restaurant process* [33,63], exhibiting a simple form of memory/path dependence. Hence, we refer to it as the Chinese restaurant process with memory (CRPM). In Figure 2 we sketch the rules of this process. Crucially, as t→∞, the random variable accounting for the number of tables m(t) has the following convergent behavior; see Proposition A5 of the Appendix D.2 for details:

$$\frac{m(t)}{\sqrt{2t}}\to 1.$$

**Figure 3:**
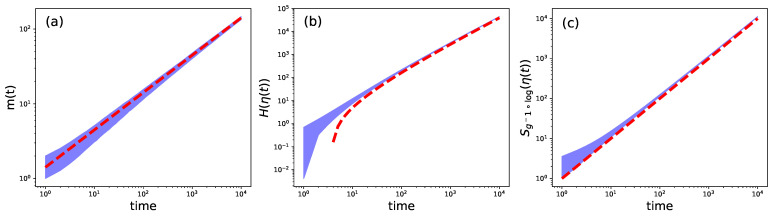
Numerical simulations for the *Chinese Restaurant process with memory*. The blue cloud represents actual numerical outcomes, dashed orange line the theoretical prediction. Time is given in arbitrary coordinates, representing a step in the process. In (**a**) we show the evolution of the amount of occupied tables against the prediction $m\left(t\right)∼\sqrt{2t}$. (**b**) The evolution of Shannon path entropy for the CRPM, being the prediction given in (11). The dashed red line shows the function $g\left(t\right)∼\frac{t}{2}\log t$. (**c**) Evolution of the generalized path entropy $S_{\Lambda }$, with Λ as defined in Equation (14). Numerical outcomes have been obtained from 1000 replicas of the whole CRPM process up to $t=10^{4}$ steps.

In Figure 3a we see that the prediction $m\left(t\right)∼\sqrt{2t}$ is quite accurate when compared to numerical simulations of the process. This property enables us to demonstrate that the CRPM we are studying is actually a CSP with compact scale $\left(\log,\frac{t}{2}\log t\right)$; see Theorem A1 of the Appendix D.3. In particular, Equation (1) is satisfied, in this particular case as:

$$\lim_{t\to \infty }\frac{1}{\frac{t}{2}\log t}\log\left(\frac{1}{p(\eta (t))}\right)=1,$$

in probability. In addition, the process is *simple*; see Theorem A2. Since we are using Λ=log, the entropy form that will arise is Shannon path entropy, by direct application of Equation (5), i.e., $S_{\Lambda }\left(\eta \left(t\right)\right)=H\left(\eta \left(t\right)\right)$, with H(η(t)) defined as:

(11) $$H\left(\eta \left(t\right)\right)=-\sum_{x(t)\in \Omega (t)}p\left(x\left(t\right)\right)\log p\left(x\left(t\right)\right).$$

It directly follows that:

(12) $$\frac{H(\eta (t))}{\frac{t}{2}\log t}\to 1.$$

Given the compact scale used, one can estimate the evolution of the size of the typical set as:

(13) $$|A\left[\epsilon_{t}\right]|∼\sqrt{\Gamma (t)},$$

where Γ is the standard Γ-function [64]. We see that the growth of the typical set as shown in Equation (13) is clearly faster than exponential. The phenomenon of *concentration of measure* [29] is clearly manifested here, as the size of the typical set vanishes in relation to the size of the whole potential set of outcomes, Ω(t). A rough estimation leads to |Ω(t)|∼Γ(t), leading to:

$$\frac{\left|A[\epsilon_{t}]\right|}{\left|\Omega (t)\right|}∼\frac{1}{\sqrt{\Gamma (t)}}\to 0,$$

despite $P(x\left(t\right)\in A\left[\epsilon_{t}\right])\to 1$.

Additionally, in Figure 3b we see that the prediction made in Equation (12) fits perfectly with the numerical realizations of the process. Note that we have shown the dependence on Shannon path entropy for the clarity in the exposition. Indeed, as pointed out above, a CSP η may have several compact scales. For example, taking the compact scale that led to Shannon entropy, (log,g(t)), with $g\left(t\right)=\frac{t}{2}\log t$, one can construct another compact scale for the CRPM by composing $g^{-1}$ (which, by assumption, exists) with both functions. In consequence, one will have a new compact scale $\left(\Lambda ,\tilde{g}\right)$, defined as:

(14) $$\Lambda \left(t\right)=\left(g^{-1}\circ \log\right)\left(t\right)∼\frac{2\log(t)}{W(2\log(t))}\;,\;\tilde{g}\left(t\right)=t,$$

where W is the positive, real branch of the *Lambert* function [64]. In Figure 3c we see that $S_{\Lambda }\left(\eta \left(t\right)\right)$ fits perfectly g(t)∼t, proving that (Λ,t) is a compact scale for the CRPM; see also Appendix D.5. We observe that this particular compact scale makes the path entropy $S_{\Lambda }$ *extensive* when applied to the CRPM.

## 3. Discussion

We demonstrated that, for a very general class of stochastic processes, which we refer to as *compact stochastic processes*, the typical set is well defined, as the probability measure tends to concentrate in clearly identifiable regions of the space of possible outcomes of the process. These processes can be path dependent, contain arbitrary internal correlations or display dynamic behavior of the phase space, showing sub- or super-exponential growth on the effective number of configurations the system can achieve. The only requirement is that there exist two functions Λ,g for which Equation (1) holds. Along the existence of the typical set, a generalized form of entropy naturally arises, from which, in turn, the cardinality of the typical set can be computed.

The existence of the typical set in systems with arbitrary phase space growth opens the door to a proper characterization, in terms of statistical mechanics, of a number of processes, mainly biological, where the number of configurations and states changes over time. In particular, it paves the path towards the statistical-mechanics-like understanding of processes showing open-ended evolution on the basis of typicality. For example, this could encompass thermodynamic characterizations of—part of—the developmental paths in early stages of embryogenesis. The existence of the typical set, even in some extreme scenarios of stochasticity and phase space behavior, leads us to the speculative hypothesis that typicality may underlie the astonishing reproducibility and precision of some biological processes. In this scenario, stochasticity would drive the system to the set of *correct* configurations—those belonging to the typical set—with high accuracy. Selection, in turn, would operate on typical sets, thereby promoting certain stochastic processes over others. More specific scenarios are nevertheless required in order to make this intriguing hypothesis more sound. Further works should clarify the potential of the proposed probabilistic framework to accommodate generalized, consistent forms of thermodynamics and explore the complications that can arise due to the loss of ergodicity that is characteristic for some of the processes that are nonetheless compatible with the above description.

Importantly, our results provide a potential starting point for an ensemble formalism for systems with sub- or super-exponential phase space growth. This opens the possibility to extend the concept of thermodynamic limit to these systems without requiring further conditions such as microscopic detailed balance, which cannot be justified in a broad range of out-of-equilibrium processes. Questions like the definition of free energies or the possible need of extensivity, which have to be answered in order to progress towards a complete and consistent thermodynamic picture remain, however, open. For tentative answers to those questions, connections to early proposals could be drawn, both on the thermodynamic grounds; see, e.g., [36,65,66], and from the perspective of entropy characterization; see, e.g., [37,44,46,53,54]. In this paper we meet an equivalence relation underlying compact scales, which allows us to transform between compact scales that do not differ too strongly, i.e., not more than by a power, without essentially changing the structure of the typical set sequence, a fact that one can utilize to give the entropic functional particular properties. For instance making the entropy associated with the compact scale extensive, as we demonstrated above for the CRPM. However, this also introduces the possibility that processes may have more than one inequivalent compact scale. We are aware that, in order to demonstrate the full potential of the theory one should aim at examples more extreme than the Chinese restaurant process we present. However, this would require an additional inquiry into the potentially hierarchic structure of potentially inequivalent compact scales that both in technical terms and conceptual terms go beyond the scope of this paper. This intriguing issue may be related to different levels of coarse graining, and deserves further investigations.

We finally point out the implications of our results for the study of information sources, given the fundamental role the typical set plays in optimal coding and data compression. The existence of the typical set in these broad class of information sources, where, simply put, the information flow is not constant, may open the possibility of new compressing strategies. These strategies could be based, for example, on properties of the specific CSP representing the information source and the compact scale Λ,g that characterize the process.

## Figures and Tables

**Figure A1:**
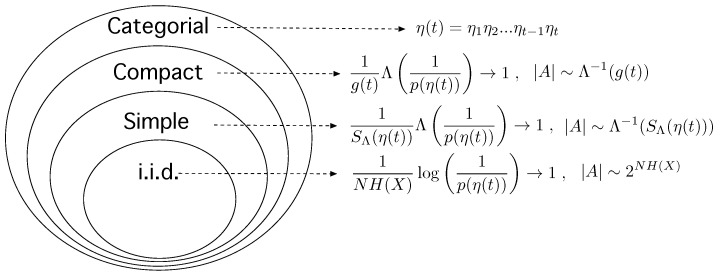
A potential hierarchy of discrete stochastic processes. The largest class would correspond to the *categorial* processes, which comprise almost anything that can be conceived as a discrete stochastic process. A subclass of categorial processes are the *compact* processes, by which the convergence condition stated in the Equation (1) of the main text holds and, therefore, a sequence of typical sets can be identified. Inside the compact processes, we identify the subclass of *simple* processes, where the sequence of typical sets can be defined in terms of a general form of entropy. Finally, the simplest subclass is the one defined by stochastic processes defined by sequences of independent, identically distributed random variables ∼X, by which the sequence of typical sets can be defined from the entropy in Shannon-like form. In turn, in i.i.d. systems, the path entropy up to time *t* can be written as *t* times the contribution of a single event [24]. Note that no assumptions of independence or stability of the sampling space are needed in the two first subclasses. Even the typical set can be consistently identified. In addition, as we will see in Appendix D, the use of Shannon entropy to characterize the sequence of typical sets is not restricted to i.i.d. systems.

## Appendix A

In this appendix we provide the mathematical details required to completely justify the results contained in the main text of the manuscript “The typical set and entropy in stochastic systems with arbitrary phase space growth”.

## Appendix B. Compact Categorial Processes

We start by providing a general framework for the processes considered in the paper. In Figure A1, below, we outline the hierarchy that our study induces over stochastic processes.

### Appendix B.1. Categorial Processes

Categorial processes η are processes that at any time *t* sample one state from a finite number of distinguishable states collected in the set $\Omega_{t}$, called the *sample space* of the process at time *t*. If we look at a discrete time line T=1,2,3,… we represent the process η up to time *t* as a sequence of random variables $\eta_{t}$, i.e.,

$$\eta \left(t\right)=\eta_{1}\eta_{2}\cdots \eta_{t-1}\eta_{t}.$$

The process η neither needs to consist of statistically independent random variables $\eta_{t}$ nor does the sample space of the process need to be constant. That is, the local sample spaces $\Omega_{t}$ of the variable $\eta_{t}$ can differ from the sample space $\Omega_{t^{\prime }}$ of another variable $\eta_{t^{\prime }}$. If we sample η(t) this provides us with a particular path:

$$x\left(t\right)=x_{1}x_{2}\cdots x_{t-1}x_{t},$$

with x(t)∈Ω(t), being Ω(t) defined as:

(A1) $$\Omega \left(t\right)=\Omega_{1}\times \Omega_{2}\times \cdots \times \Omega_{t-1}\times \Omega_{t},$$

in other words, the Cartesian product of all local sample spaces $\Omega_{t^{\prime }}$ up to time *t*.

In principle it could be that also the sample space $\Omega_{t}$ depends on the path x(t−1) the process has taken up to time t−1. In consequence, Ω(t) could contain many paths that are not possible for the process η and, therefore, have zero probability of being sampled. However, in such a case we can always consider the non-empty subset $\overset{˚}{\Omega }\left(t\right)\subseteq \Omega \left(t\right)$, which contains all paths x(t), and only those paths of η, with p(x(t))>0.

**Definition** **A1.**
*Let η be a categorial process. We call p(x(t)) the probability of paths x(t)∈Ω(t) to be sampled by η and, then, we call:*

(A2)
$$\overset{˚}{\Omega }\left(t\right)=\left\{x\left(t\right)\in \Omega \left(t\right)\;:\;p\left(x\left(t\right)\right)>0\right\},$$

*the **well formed interior** of Ω(t) or the **path sample space** of the process.*

Let us call $\Omega_{t}\left[x\left(t-1\right)\right]$ the set of states of $\Omega_{t}$ that can be sampled at time *t provided that* our trajectory up to time t−1 was x(t−1). The set of potential states that can be visited at time *t*, will then be:

$$\Omega_{t}=\underset{x\left(t-1\right)\in \overset{˚}{\Omega }\left(t-1\right)}{⋃}\Omega_{t}\left[x\left(t-1\right)\right],$$

i.e., $\Omega_{t}$ contains all states the process could possibly sample at time *t* after all possible histories the process could have sampled up to time t−1. In this way we can always assume that we can find Ω(t) and its well formed interior $\overset{˚}{\Omega }\left(t\right)$ by pruning all ill formed sequences from $\Omega_{t}\times \overset{˚}{\Omega }\left(t-1\right)$. All information on how the sample space $\Omega_{t}$ gets sampled then solely resides in the hierarchy of transition probabilities $p(x_{t}|x\left(t-1\right))$, where $x_{t}\in \Omega_{t}$ and x(t−1)∈Ω(t−1). We therefore get:

$$\overset{˚}{\Omega }\left(t\right)=\left\{x\left(t\right)\in \Omega_{t}\times \overset{˚}{\Omega }\left(t-1\right)\;:\;p\left(x_{t}|x\left(t-1\right)\right)>0\right\}.$$

In the context of categorial processes, p(x(t)) is clearly a monotonic decreasing function in time bounded below by zero, p(x(t))≥0. In consequence, $\lim_{t\to \infty }p\left(x\left(t\right)\right)$ converges for all possible paths x(t) of the process η. This also means that for almost all paths the probability $\lim_{t\to \infty }p\left(x\left(t\right)\right)=0$ even though some finite number of paths could have non-zero probabilities even in the limit t→∞, although convergence is guaranteed (Unlike for processes, for *systems*, e.g., particles in a box, it is not guaranteed that adding a new particle, as analog of to a new sample step, p(x(t))≤p(x(t−1). In consequence, for systems, we cannot guarantee convergence of p(x(t)) as the system size t→∞). After this general description of categorial processes, we can start by characterizing the subclass of them we are interested in. In the following we provide the condition of compactness that we impose to categorial processes in order to ensure the existence of a typical set.

**Definition** **A2.**
*Let η be a categorial process η and let η(t) denote the process up to time t and Ω(t) denote the path sample space of η(t) (as discussed above). Let us consider pairs of functions (Λ,g), such that *Λ* is a twice continuously differentiable, strictly monotonic increasing and strictly concave function on the interval [1,∞) with $\lim_{t\to \infty }\Lambda \left(t\right)=\infty$ and Λ(1)=0; and g is a twice continuously differentiable strictly monotonically increasing function on the interval [0,∞). If we can **associate** such a pair of functions (Λ,g) with the process η, such that:*

$$\lim_{t\to \infty }\frac{1}{g(t)}\Lambda \left(\frac{1}{p(\eta (t))}\right)\;=\;1,$$

*(in probability), then we call the process η **compact** in (Λ,g), and (Λ,g) a **compact scale** of η.*

We will call these processes *Compact Stochastic Processes* (CSP). Note that compact scales (Λ,g) associated to a given CSP need not be unique (The fact that we can find different pairs of functions, (Λ,g), in which a process η is compact, leads to questions related to equivalence relations on the space of pairs, (Λ,g). As it turns out, following up the idea of typical sets, that we are to explore below, a process η induces an equivalence relation on this space, partitioning the space into monads of equivalent compact scales, (Λ,g), of the process. At the same time this means that there exist inequivalent compact scales, which can be thought of as different “scales of resolution” to look at a process.).

### Appendix B.2. Properties of *Λ*

The next step is to check what kind of functions Λ can be expected when dealing with CSPs. To that end, we will impose a condition to the CSP, namely, that the CSP is *filling*. From this, very mild, condition, we will then check which functions enable the convergence criteria to be fullfilled. First of all, we need to introduce some technical terms.

**Definition** **A3.**
*Let η be a CSP in (Λ,g). Let $\epsilon_{1},\ldots ,\epsilon_{t},\ldots$ be a non-increasing sequence of real numbers such that $\lim_{t\to \infty }\epsilon_{t}=0$, from which one can construct a sequence of typical sets $A\left[\epsilon_{1}\right]\ldots A\left[\epsilon_{t}\right]$ of the respective sample spaces Ω(1)…Ω(t) such that $P(x\left(t\right)\in A\left[\epsilon_{t}\right])\to 1$. We define the upper and lower **typical ratios**, $r_{\pm }\left(t\right)$, in (Λ,g) as:*

$$r_{\pm }\left(t\right)=\frac{\Lambda^{-1}\left(\left(1\pm \epsilon_{t}\right)g\left(t\right)\right)}{\Lambda^{-1}\left(g(t)\right)},$$

*where $\Lambda^{-1}$ is the inverse function of *Λ*, which exists due to the strict monotonicity of *Λ*.*

Note that, by construction, $r_{+}\left(t\right)\geq 1$ and $r_{-}\left(t\right)\leq 1$.

**Definition** **A4.**
*We call a CSP η **filling** in (Λ,g) if its typical ratios have the property $\lim_{t\to \infty }r_{+}\left(t\right)$=∞ and $\lim_{t\to \infty }r_{-}\left(t\right)=0$.*

We can now say something about the shape of functions Λ.

**Proposition** **A1.**
*If η is a filling CSP in (Λ,g), then it follows that:*

$$\lim_{z\to \infty }\frac{\Lambda (\lambda z)}{\Lambda (z)}=1,$$

*for all λ>0.*

**Proof.** We note that for filling CSP η it is true that:

$$\frac{1}{g(t)}\Lambda \left(r_{\pm }\left(t\right)\Lambda^{-1}\left(g\left(t\right)\right)\right)\to 1,$$

as t→∞. We can rewrite $z_{t}=\Lambda^{-1}\left(g\left(t\right)\right)$ and for λ>1 we find a $t_{0}$ such that $\lambda =r_{+}\left(t_{0}\right)$. Therefore for all $t>t_{0}$ we find:

$$1\leq \frac{\Lambda (\lambda z_{t})}{\Lambda (z_{t})}\leq \frac{\Lambda (r_{+}\left(t\right)z_{t})}{\Lambda (z_{t})}\to 1.$$

The other case, 0<λ<1, we prove analogously using $r_{-}$ instead of $r_{+}$, and the proposition follows. □

To get an idea which kind of functions satisfy this condition we can look at the following example:

**Proposition** **A2.**
*For any c>0 the function $\Lambda \left(z\right)=\log{\left(z\right)}^{c}$ has the property $\lim_{z\to \infty }\Lambda \left(\lambda z\right)/$Λ(z)=1 for all λ>0.*

**Proof.** We can prove this by direct computation, i.e.,

$$\begin{array}{ccc} \frac{\Lambda (\lambda z)}{\Lambda (z)} & = & {\left(\frac{\log(\lambda z)}{\log(z)}\right)}^{c}\\ & = & {\left(\frac{\log(\lambda )+\log(z)}{\log(z)}\right)}^{c}\\ & = & {\left(1+\frac{\log(\lambda )}{\log(z)}\right)}^{c}. \end{array}$$

Since log(z)→∞, one can easily read from the last line that the example family of functions Λ fulfills the proposition. □

In general we can say that candidates for Λ of filling CSPs are of the form $\Lambda \left(z\right)=c\log{\left(z\right)}^{d}$ for some positive constants *c* and *d* or even slower growing functions of the form:

$$\Lambda =c_{1}\log{\left(1+c_{2}\log{\left(1+c_{3}\log{\left(\cdots \right)}^{d_{3}}\right)}^{d_{2}}\right)}^{d_{1}}.$$

where $c_{1},\ldots$ and $d_{1},\ldots$ are positive, real valued constants.

## Appendix C. Properties of the Generalized Entropies

The generalized entropy associated to a CSP η in (Λ,g) is defined as:

**Definition** **A5.**
*Let η be a CSP in (Λ,g), then we call the measure, $S_{\Lambda }$ of η(t), defined as:*

(A3)
$$S_{\Lambda }\left(\eta \left(t\right)\right)=\sum_{x(t)\in \Omega (t)}p\left(x\left(t\right)\right)\Lambda \left(\frac{1}{p(x(t))}\right),$$

*a **generalized path entropy** associated with η. Note that for a set B⊂Ω(t) the generalized entropy measure, $S_{\Lambda }\left(B\right)$, is given by $S_{\Lambda }\left(B\right)=\sum_{x(t)\in B}p\left(x\left(t\right)\right)\Lambda \left(1/p(x(t))\right)$.*

In the following proposition we see that the above defined entropy satisfies three of the four Shannon–Khinchin axioms for an entropy measure [62] (**SK1**, **SK2**, **SK3**). The fourth axiom (**SK4**) is not generally satisfied.

**Proposition** **A3.**
*The entropy functional $S_{\Lambda }$ defined in Equation (A3) satisfies the first three of the four Shannon–Khinchin axioms for an entropy measure as formulated in [62]:*

**SK1** *$S_{\Lambda }$ is a continuous function only depending on the probabilities p(x(t)).*
**SK2** *$S_{\Lambda }$ is maximized if (∀p(x(t))) $p\left(x\left(t\right)\right)=\frac{1}{\left|\Omega (t)\right|}$, i.e., equiprobability.*
**SK3** *If p(x(t))=0, then: $p\left(x\left(t\right)\right)\Lambda \left(\frac{1}{p(x(t))}\right)=0$, i.e., events with zero probability have no contribution to the entropy.*

**Proof.** To demonstrate **SK1**, it is enough to observe that $S_{\Lambda }$ is only function of the probabilities and to take into account that, by assumption, $\Lambda \in C^{2}$, therefore, $S_{\Lambda }$ continuous. To demonstrate **SK2**, we need to maximize the functional ψ, defined as:

$$\psi =S_{\Lambda }\left(\eta \left(t\right)\right)-\alpha \left(\sum_{x(t)\in \Omega (t)}p\left(x\left(t\right)\right)-1\right),$$

where α is a Lagrangian multiplier implementing the normalization constraint. Maximizing ψ with respect to a p(x(t)) yields:

$$0=\frac{\partial \psi }{\partial p(x(t))}=\Lambda \left(z\right)-z\Lambda^{\prime }\left(z\right)-\alpha ,$$

where z=1/p(x(t)) and $\Lambda^{\prime }$ is the first derivative of Λ. Note that if the equation does not depend explicitly on x(t) and if it has a unique solution then the proposition is proved, since all p(x(t)) have the same value. To see that a unique solution exists we need to show that $f\left(z\right)=\Lambda \left(z\right)-z\Lambda^{\prime }\left(z\right)$ is strictly monotonic. To see that, it is enough to note that the first derivative of *f* is given by $f^{\prime }\left(z\right)=-z\Lambda^{\prime \prime }\left(z\right)>0$, since, by definition of compactness, $\Lambda \in C^{2}$ and strictly concave; and therefore $\Lambda^{\prime \prime }\left(z\right)<0$. Finally, to demonstrate that $S_{\Lambda }$ satisfies **SK3** we apply the l’Hopital rule. First, by defining y=1/z, one has that:

$$\lim_{z\to 0}z\Lambda \left(\frac{1}{z}\right)=\lim_{y\to \infty }\frac{1}{y}\Lambda \left(y\right).$$

Then, considering that, by definition, $\Lambda \in C^{2}$ is a strictly growing and concave function, one is led, after application to the l’Hopital rule for the limit, to:

$$\lim_{y\to \infty }\frac{1}{y}\Lambda \left(y\right)=\lim_{y\to \infty }\Lambda^{\prime }\left(y\right)=0,$$

thereby concluding the proof. □

We observe that **SK3** enables us to safely perform the sum for the entropy over the whole set of paths Ω(t), since $\sum_{x\left(t\right)\in \overset{˚}{\Omega }\left(t\right)}\left(...\right)=\sum_{x(t)\in \Omega (t)}\left(...\right)$.

## Appendix D. The Chinese Restaurant Process

We now turn to analyzing the version of the Chinese restaurant process with memory (CRPM) discussed in the main body of the paper. The version presented here is a variation of the standard Chinese Restaurant process as found in [33,63].

### Appendix D.1. Definition and Basics

Suppose a restaurant with an infinite set of tables $m_{1},\ldots ,m_{n},\ldots$ each with infinite capacity. The first customer enters and sits at the first table $m_{1}$. The second customer now has a choice to also sit down at the first table $m_{1}$ together with the first customer or to choose a free table $m_{2}$, each with probability 1/2. Let m(t−1) be the number of occupied tables at t−1. If the *t*’th customer finds that m(t−1) tables are already occupied by some guests, then again the customer will choose one of the non-empty tables:

$$m_{1},\ldots ,m_{m(t-1)},$$

each with probability 1/(m(t−1)+1), in which case m(t)=m(t−1), or the next empty table $m_{m(t-1)+1}$, also with probability 1/(m(t−1)+1). In this latter case m(t)=m(t−1)+1. The key point is therefore the number of occupied tables m(t). We observe that the amount of occupied tables can be rewritten as a stochastic recurrence:

(A4) m(t+1)=m(t)+ζ(m(t)),

where ζ(m(t)) is a random variable by which:

$$p\left(\zeta \left(m\left(t\right)\right)=0\right)=\frac{m(t)}{m(t+1)}\;,\;p\left(\zeta \left(m\left(t\right)\right)=1\right)=\frac{1}{m(t+1)}.$$

Clearly,

$$m\left(t\right)=1+\sum_{t^{\prime }\leq t}\zeta \left(m\left(t\right)\right),$$

is a non-decreasing function in *t*. Now let us define $M_{k}\left(t\right)$ as a random variable taking values uniformly at random over the set $m_{1},\ldots ,m_{k},m_{k+1}$ at time *t*. The sequence of random variables accounting describing the CRPM η(t) can be written as:

$$\eta \left(t\right)=M_{1}\left(1\right),M_{1}\left(2\right),M_{m(2)}\left(3\right),\ldots ,M_{m(t)}\left(t+1\right),\ldots ,$$

leading to paths x(t) of the kind:

$$x\left(t\right)=m_{1},m_{1},m_{2},m_{1},m_{2},m_{3},m_{2},m_{2},m_{1},m_{3},\ldots$$

We emphasize that *the tables visited are distinguishable and can be visited repeatedly*. The CRPM however does not fill up Ω(t), i.e., there exist elements in $\Omega \left(t\right)=\times_{t^{\prime }=1}^{t}\Omega_{t^{\prime }}$, that are not potential paths of the CRP. This includes all sequences that select a table $m_{i}$ without ever having chosen some table $m_{j}$, with j<i before. For example, the path $x\left(t\right)=\left(m_{1},m_{2},m_{1},m_{2},m_{3},m_{1},m_{5},m_{4},m_{3},\cdots \right)$ is not possible, because $m_{5}$ is chosen before $m_{4}$. The CRPM therefore gives us the opportunity to introduce sampling spaces conditional to a particular well formed path. In this particular case, it is enough to observe that the sampling space some well formed path x(t)∈Ω(t−1)*sees* at time *t* is given by:

(A5) $$\Omega_{t}\left[x\left(t-1\right)\right]=\left\{m_{1},m_{2},\cdots ,m_{m[x](t-1)]+1}\right\},$$

where m[x](t−1) is the number of different tables the well formed CRPM path x(t−1) has sampled at time t−1. By convention, we define m[x](1)=1.

### Appendix D.2. Statistics of the CRPM

We start computing the probability of a particular path $x\left(t\right)\in \overset{˚}{\Omega }\left(t\right)$.

**Proposition** **A4.**
*Let process η(t) be the CRPM and $x\left(t\right)\in \overset{˚}{\Omega }\left(t\right)$ be a given path of the process up to time t. Let $m\left[x\right]\left(t^{\prime }\right)$ be the number of occupied tables in the restaurant associated with the path $x(t^{\prime })$ at time $t^{\prime }$. Then the probability to observe the particular sequence of tables, p(x(t)) is given by:*

$$p\left(x\left(t\right)\right)=\prod_{t^{\prime }=1}^{t}{\left(m\left[x\right]\left(t^{\prime }\right)+1\right)}^{-1}.$$

**Proof.** The proposition follows from direct calculation. □

Now we will see that the sequence corresponding to the number of occupied tables converges to a tractable functional form.

**Proposition** **A5.**
*Given the sequence of occupied tables of the CRPM m(1),…,m(t) as defined in Equation (A4), then:*

$$\frac{m(t)}{\sqrt{2t}}\to 1,$$

*in probability.*

**Proof.** Consider the random variable $\Delta t_{k}$ denoting the amount of steps by which m(t)=k. $\Delta t_{k}$ is a geometric random variable with associated law:

$$p\left(\Delta t_{k}=i\right)={\left(1-\frac{1}{k}\right)}^{i-1}\frac{1}{k}\;,\;\langle \Delta t_{k}\rangle =k\;,\;\sigma^{2}\left(\Delta t_{k}\right)=k^{2}-k.$$

Now we construct a new set of renormalized random variables, $\delta t_{1},\ldots ,\delta t_{k}$ as:

$$\delta t_{k}\equiv \frac{\Delta t_{k}}{k}\;,\;\langle \delta t_{k}\rangle =1\;,\;\sigma^{2}\left(\delta t_{k}\right)=1-\frac{1}{k}.$$

In that context, the sum of $\delta t_{1},\ldots ,\delta t_{k}$ is the sum of *k* random variables with mean 1 and $\sigma^{2}<1$. Therefore, there exists a monotonically increasing function, μ(t) by which, by the law of large numbers, for each pair ϵ,δ<0, there exists $t_{0}$ such that, for $t>t_{0}$:

$$P\left(\left|\frac{\sum_{k\leq m(t)}\delta t_{k}}{\mu (t)}-1\right|>\epsilon \right)<\delta .$$

Notice that, in this setting, we have that the deviations behave close to a discrete random walk centered at 0 and with step length $\sigma^{2}<1$. Since for all $\delta t_{k}$, $\langle \delta t_{k}\rangle =1$:

$$\frac{m(t)}{\mu (t)}\to 1\;,\;\left(\mathrm{in}\;\mathrm{probability}\right)\;,$$

and, since, by construction:

$$\sum_{k\leq \mu (t)}k\delta t_{k}=t,$$

one has that:

$$\mu \left(t\right)∼\sqrt{2t}.$$

where “∼” means asymptotically equivalent, as we wanted to demonstrate. □

### Appendix D.3. The Typical Set of the CRP

**Theorem** **A1.**
*The CRPM is compact with compact scale $\left(\Lambda ,g\left(t\right)\right)=\left(\log,\frac{t}{2}\log t\right)$.*

**Proof.** We need to demonstrate that:

$$\frac{1}{\frac{t}{2}\log t}\log\left(\frac{1}{p(\eta (t))}\right)\to 1\;,\;\;\left(\mathrm{in}\;\mathrm{probability}\right).$$

We first note that, according to the definition of the sequence of the number of occupied tables given in Equation (A4), and the statement of Proposition A4, one can rewrite the logarithmic term of the condition for compactness as:

$$\log\left(\frac{1}{p(\eta (t))}\right)=\sum_{t^{\prime }\leq t}\log\left(m\left(t^{\prime }\right)\right).$$

Now, let us define a new random variable z(t) as follows:

z(t)=m(t)−μ(t),

with $\mu \left(t\right)=\sqrt{2t}$. Notice that, according to Proposition A5, we have that:

$$\frac{z(t)}{\mu (t)}\to 0\;,\;\left(\mathrm{in}\;\mathrm{probability}\right).$$

We can then rewrite condition of compactness, with $g\left(t\right)=\sum_{t^{\prime }\leq t}\log\left(\mu \left(t\right)\right)$, as:

$$\begin{array}{ccc} \frac{1}{\sum_{t^{\prime }\leq t}\log\left(\mu \left(t^{\prime }\right)\right)}\log\left(\frac{1}{p(\eta (t))}\right) & = & \frac{1}{\sum_{t^{\prime }\leq t}\log\left(\mu \left(t^{\prime }\right)\right)}\sum_{t^{\prime }\leq t}\log\left(m\left(t^{\prime }\right)\right)\\ & = & \frac{1}{\sum_{t^{\prime }\leq t}\log\left(\mu \left(t^{\prime }\right)\right)}\sum_{t^{\prime }\leq t}\log\left(\mu \left(t^{\prime }\right)+z\left(t^{\prime }\right)\right)\\ & = & \frac{1}{\sum_{t^{\prime }\leq t}\log\left(\mu \left(t^{\prime }\right)\right)}\sum_{t^{\prime }\leq t}\log\left(\mu \left(t^{\prime }\right)\right)+\log\left(1+\frac{z(t^{\prime })}{\mu (t^{\prime })}\right)\\ & = & 1+\frac{1}{\sum_{t^{\prime }\leq t}\log\left(\mu \left(t^{\prime }\right)\right)}\sum_{t^{\prime }\leq t}\log\left(1+\frac{z(t^{\prime })}{\mu (t^{\prime })}\right). \end{array}$$

It remains to see that the second term of the sum goes to 0. Clearly, by Proposition A5:

$$\frac{\log\left(1+\frac{z(t)}{\mu (t)}\right)}{\log(\mu (t))}\to 0\;,\;\;\left(\mathrm{in}\;\mathrm{probability}\right)\;.$$

Consequently:

$$\frac{1}{\sum_{t^{\prime }\leq t}\log\left(\mu \left(t^{\prime }\right)\right)}\log\left(\frac{1}{p(\eta (t))}\right)\to 1\;,\;\;\left(\mathrm{in}\;\mathrm{probability}\right)\;.$$

Finally, we need to compute the asymptotic form of $\sum_{t^{\prime }\leq t}\log\left(\mu \left(t^{\prime }\right)\right)$. Observing that we have a Riemann sum, one can consider:

$$\sum_{t^{\prime }\leq t}\log\left(\mu \left(t^{\prime }\right)\right)∼\int^{t}\log\left(\sqrt{2t^{\prime }}\right)dt^{\prime }∼\frac{t}{2}\log t,$$

thus concluding the proof. □

### Appendix D.4. The Entropy of the CRP

We demonstrate here that the CRPM is simple. In consequence, the typical set can be computed as a function of the entropy. In that case, one can show that a suitable choice is Λ=log, chosen for the sake of simplicity, implying that the associated entropy is Shannon path entropy. However, we emphasize that this choice is not unique. Given a different choice of *g*, one could have another Λ by which the process is also simple and, therefore, the typical set could be defined through another form of entropy. We briefly comment on this point in the next section, sketching how another potential pair (Λ,g) functions would work as well.

**Theorem** **A2.**
*The CRPM is simple in $\left(\Lambda ,g\left(t\right)\right)=\left(\log,\frac{t}{2}\log t\right)$.*

**Proof.** Since the CRPM is compact with compact scale $\left(\Lambda ,g\left(t\right)\right)=\left(\log,\frac{t}{2}\log t\right)$, we know that there is a typical localizer sequence $\epsilon_{1},\ldots ,\epsilon_{t},\ldots$ with associated $\delta_{1},\ldots ,\delta_{t},\ldots$, such that $\lim_{t\to \infty }\epsilon_{t}=\lim_{t\to \infty }\delta_{t}=0$. The paths belonging to the typical set $A[\epsilon_{t}]$, are those satisfying:

$$e^{-\left(1+\epsilon_{t}\right)\frac{t}{2}\log t}\leq p\left(x\left(t\right)\right)\leq e^{-\left(1-\epsilon_{t}\right)\frac{t}{2}\log t}.$$

In addition, the measure associated to the typical set $A[\epsilon_{t}]$ is given by:

$$P\left(x\left(t\right)\in A\left[\epsilon_{t}\right]\right)\geq 1-\delta_{t}.$$

In consequence,

$$\begin{array}{ccc} \left(1-\epsilon_{t}\right)\left(1-\delta_{t}\right)\frac{t}{2}\log t & \leq & -\sum_{x\left(t\right)\in A\left[\epsilon_{t}\right]}p\left(x\left(t\right)\right)\log p\left(x\left(t\right)\right)\\ & \leq & \left(1+\epsilon_{t}\right)\left(1-\delta_{t}\right)\frac{t}{2}\log t. \end{array}$$

Now let us consider that the complement of the typical set $\Omega \left(t\right)\setminus A\left[\epsilon_{t}\right]$, whose measure is:

$$P\left(x\left(t\right)\in \Omega \setminus A\left[\epsilon_{t}\right]\right)\leq \delta_{t},$$

is completely populated by those paths by which:

$$x\left(t\right)\in \Omega \setminus A\left[\epsilon_{t}\right]\;,\;p\left(x^{*}\left(t\right)\right)=\min_{\overset{˚}{\Omega }\left(t\right)}\left\{p\left(x\left(t\right)\right)\right\},$$

therefore, for all paths $x\left(t\right)\in \overset{˚}{\Omega }\left(t\right)$:

$$-\log p\left(x\left(t\right)\right)\leq -\log p^{*}\left(x\left(t\right)\right).$$

The least probable path is the one that increases the number of tables at every step, having a probability $p^{*}\left(x\left(t\right)\right)$ of:

$$p^{*}\left(x\left(t\right)\right)=\frac{1}{t!},$$

leading to $-\log p^{*}\left(x\left(t\right)\right)∼t\log t$, thanks to the Stirling approximation [64]. In that context:

$$-\sum_{x\left(t\right)\in \Omega \left(t\right)\setminus A\left[\epsilon_{t}\right]}p\left(x\left(t\right)\right)\log p\left(x\left(t\right)\right)\leq \delta t\log t.$$

Collecting the above reasoning, and by observing that the defined entropy is actually the *Shannon path entropy*, $S_{\Lambda }\left(\eta \left(t\right)\right)=H\left(\eta \left(t\right)\right)$ [24]:

$$H\left(\eta \left(t\right)\right)=-\sum_{x(t)\in \Omega (t)}p\left(x\left(t\right)\right)\log p\left(x\left(t\right)\right),$$

one has that:

$$\begin{array}{ccc} \left(1-\epsilon_{t}\right)\left(1-\delta_{t}\right)\frac{t}{2}\log t & \leq & H(\eta (t))\\ & \leq & \left(1+\epsilon_{t}\right)\left(1-\delta_{t}\right)\frac{t}{2}\log t+\delta_{t}t\log t. \end{array}$$

In consequence, by defining $g\left(t\right)=\frac{t}{2}\log t$, one is led to:

$$\left(1-\delta_{t}\right)\left(1-\epsilon_{t}\right)\leq \frac{H(\eta (t))}{g(t)}\leq \left(1-\delta_{t}\right)\left(1+\epsilon_{t}\right)+2\delta_{t}.$$

Since we know that $\lim_{t\to \infty }\epsilon_{t}=\lim_{t\to \infty }\delta_{t}=0$ we conclude that:

$$\frac{H(\eta (t))}{g(t)}\to 1\;,$$

as we wanted to demonstrate. □

### Appendix D.5. A Different Compact Scale (*Λ*,G) for the CRP

We finally briefly comment how another compact scale made of different (Λ,g) functions could be used to characterize the typical set and the generalized entropy of the CRP. We avoid the technical details, for the sake of simplicity. We start computing the inverse function of *g*, $g^{-1}$:

$$g^{-1}\left(z\right)\approx \frac{2z}{W(2z)},$$

where W is the *Lambert* function [64], where only the positive, real branch is taken into account. Then we compose it with the log function, thereby defining a new function Λ as:

(A6) $$\Lambda \left(z\right)=\left(g^{-1}\circ \log\right)\left(z\right)∼\frac{2\log(z)}{W(2\log(z))}.$$

We observe that Λ as above defined is a strictly growing, concave function with continuous second derivatives. Clearly, $\left(g^{-1}\circ g\right)\left(t\right)∼t$. Therefore, as a direct consequence of Theorem A1, the CRPM is compact in (Λ,t):

$$\frac{1}{g^{-1}\left(\frac{t}{2}\log t\right)}\left(g^{-1}\circ \log\right)\left(\frac{1}{p(\eta (t))}\right)=\frac{1}{t}\Lambda \left(\frac{1}{p(\eta (t))}\right)\to 1.$$

(in probability). In consequence, the size of the typical set and the typical probabilities can be approximated from the following generalized entropy $S_{\Lambda }$:

(A7) $$S_{\Lambda }\left(\eta \left(t\right)\right)=\sum_{x(t)\in \Omega (t)}p\left(x\left(t\right)\right)\frac{2\log\left(\frac{1}{p(x(t))}\right)}{W\left[2\log\left(\frac{1}{p(x(t))}\right)\right]}.$$

where we explicitly wrote it in terms the functional form of $\Lambda =g^{-1}\circ \log$ as defined in Equation (A6).
